# Do Terminally Ill Cancer Patients’ Self-Assessments and Nurses’ Assessments Agree on Cancer-Related Fatigue? A Cross-Sectional Study

**DOI:** 10.1089/pmr.2024.0064

**Published:** 2024-12-23

**Authors:** Mizuki Matsuda, Hideyuki Honma, Yu Koyama, Yuuka Kashiwagi, Mika Hattori, Yoshifumi Matsumoto, Yasuo Saijyo, Sayuri Sakai

**Affiliations:** ^1^ Graduate School of Health Sciences, Department of Nursing, Doctoral Program, Niigata University, Niigata, Japan.; ^2^ Department of Palliative Care, Niigata Cancer Center Hospital, Niigata, Japan.; ^3^ Graduate School of Health Sciences, Niigata University, Niigata, Japan.; ^4^ Department of Nursing, Niigata Prefectural Shibata Hospital, Niigata, Japan.; ^5^ Niigata Medical Association, Niigata, Japan.; ^6^ Kounan Family Clinic, Niigata, Japan.; ^7^ Department of Medical Oncology, Niigata University Graduate School of Medical and Dental Sciences, Niigata, Japan.

**Keywords:** affective fatigue, assessment, cancer-related fatigue, nurse assessment, terminal cancer

## Abstract

**Background:**

Cancer-related fatigue (CRF) is a distressing symptom in patients with terminal cancer. It is often under-assessed or remains unrecognized among health care professionals due to difficulties in identifying symptoms. However, fatigue ratings have not been studied extensively in incurable, terminally ill cancer patients with palliative intent or in nurses who provide daily care in palliative care teams or units.

**Objective:**

This study examined the agreement between nurses’ assessments and terminally ill cancer patients’ self-assessments of CRF.

**Methods:**

The correlation between patients’ scores on the Cancer Fatigue Scale (CFS) and nurses’ scores on the Japanese version of the Support Team Assessment Schedule (STAS-J) was matched in patient–nurse pairs. Paper versions of the questionnaires were answered by the patients and nurses at the time of the temperature check.

**Results:**

Twenty-eight participants in 14 pairs with valid responses were included. There were 10 cases (71.4%) of agreement between the patients’ and nurses’ assessments of fatigue via the CFS and STAS-J, respectively. Among the four cases (28.6%) of incongruence, two (14.3%) were underestimated, and two were overestimated. Significant correlations were observed between the STAS-J and physical fatigue (rs = 0.66, *p* < 0.01), but total fatigue (rs = 0.47, *p* = 0.09), affective fatigue (rs= −0.09, *p* = 0.75), and cognitive fatigue (rs = 0.52, *p* = 0.06) showed no significant correlation.

**Conclusion:**

Differences were primarily observed in affective fatigue; therefore, nurses must carefully consider affective fatigue when assessing fatigue in patients with terminal cancer.

## Introduction

Cancer-related fatigue (CRF) is among the most prevalent and devastating cancer symptoms worldwide. The reported prevalence rates range from 61% to 80% in patients actively receiving chemotherapy or radiotherapy and increase further with the progression of symptoms.^1–4^ The National Comprehensive Cancer Network defines CRF as “a distressing, persistent, subjective sense of physical, emotional, and/or cognitive tiredness or exhaustion related to cancer and/or cancer treatment not proportional to recent activity and interferes with usual functioning.”^5^ Fatigue has a significant negative impact on physical and psychological functioning, which limits the patients’ freedom of activity, resulting in a decrease in treatment satisfaction and quality of life (QoL).^6–8^ In particular, individuals in the terminal phase of cancer experience numerous physical and psychological symptoms, and this phase is more distressing owing to fatigue than other symptoms, such as pain or depression.^7,8^ In a survey of the bereaved families of patients with cancer in Japan, 28.7% and 28.1% reported pain and dyspnea, respectively, whereas CRF accounted for 30.7%. It was the most frequent distress symptom in the week before death.^9^

However, patients with cancer often fail to communicate with their doctors about fatigue because they believe it to be an untreatable symptom that one has to endure as a normal part of cancer and its treatment.^10^ In addition, fatigue is a subjective and multidimensional symptom,^11^ and medical professionals can only know about patients’ fatigue indirectly from what the patient communicates verbally. It also remains hidden in various symptoms, making it difficult to understand its severity.^12^ This can result in negative patient outcomes. In particular, failure to perceive or communicate patients’ fatigue prevents medical professionals from paying attention to this symptom, thereby potentially impacting care for them. Proper assessment of fatigue is the first step toward alleviating and treating fatigue; however, differences in perceptions and underestimation between patients undergoing chemotherapy and oncologists and nurses impact patients’ daily life.^13,14^

Terminally ill cancer patients often receive assistance with activities of daily living care from nurses, and symptom data are routinely collected by the nurses. Nurses play a particularly important role in early detection of fatigue and reporting symptoms, but fatigue ratings have not been studied extensively in incurable, terminally ill cancer patients with palliative intent or in nurses who provide daily care in palliative care teams or units. This is the first observational study on fatigue congruence in patients with terminal cancer and nurses’ perceptions. In this study, we analyzed terminally ill cancer patient reports and those of nurses belonging to palliative care or palliative care teams reporting the severity of fatigue in patients. The current study compares patient and nurse reporting of severity and examines the rate of patient–nurses agreement or disagreement and factors that may be associated with the discrepancy.

## Materials and Methods

### Study design and population

A cross-sectional study was conducted among patients with cancer receiving palliative care at Niigata Cancer Center Hospital, Japan. The participants were recruited between October 2022 and March 2023. Terminally ill cancer patients who made ambulatory visits at the palliative care clinic and inpatients in the palliative care unit were enrolled in the study if they met the following inclusion criteria: 18 years or older, informed of their cancer diagnosis, aware of their palliative status, in the terminal phase as determined by the oncologist, received hospice care, were able to complete the questionnaire, and did not have a severe mental or cognitive disorder. No restrictions were applied to patient selection in terms of the histological tumor type. Nurses affiliated with the palliative care team or palliative care unit were enrolled in the study.

Paper versions of the questionnaires were distributed to the patients and nurses by the researcher at the time of the temperature check on the day of distribution. To identify each nurse–patient pair, the questionnaires were numbered 1, 2, 3, etc., for both the patients and the nurses so that the patient and the receiving nurse pair had the same number.

When planning the research protocol, the sample, G*Power with an effect size of 0.3, alpha error of 0.05, a test power (1-β) of 0.8, and an analysis of the comparison test between two groups, two-tailed, we found that the necessary sample size was 30 patients and 30 nurses, with a total of 60.

### Data collection

Patient information included hospitalization period and period of palliative care department visits, while nurse information included years of service in specialized palliative care and previous experience with the pair’s patient. The following assessment tools were used: The Cancer Fatigue Scale (CFS),^11^ and the Support Team Assessment Schedule in Japan (STAS-J).^15^

The CFS is a 15-item self-rating scale developed by Okuyama et al. to assess fatigue in patients with cancer.^11^ This scale consists of three subscales assessing the physical, affective, and cognitive aspects of fatigue, which we call the physical, affective, and cognitive subscales, respectively. It has been tested for reliability and validation. Patients are asked to circle a number that describes their current state on a scale of 1 (not at all) to 5 (very much). Total fatigue is calculated as the sum of these aspects. The maximum total score is 60, with higher scores indicating more severe fatigue. The cut-off value is 19 points, and individuals with a score of 19 points or more are considered to have severe fatigue, which is an obstacle to activities of daily living.^16^ The validity and reliability of this scale were confirmed in a developmental study.^11^ Only patients responded to this scale.

The STAS is a proxy-rated questionnaire for the comprehensive monitoring of physical and psychological symptoms and socialization issues in patients with cancer.^17^ It has nine core items: pain control, symptom control, patient anxiety, family anxiety, patient insight, family insight, communication between patient and family, communication between professionals, and communication from professionals to patient and family. Each item receives a score from 0 (best) to 4 (worst) based on the item definition. From the perspective of health care workers, a high score represents a high level of patient distress. The common scoring criterion for each question is that a score of two or more indicates a problem requiring care.^18^ The STAS is useful for assessing difficulties and identifying the need for improvement in palliative settings.^15^ The reliability and validity of the Japanese version of the scale have been well established.^15,19^ Only nurses responded to this scale. Currently, there is no multidimensional scale to evaluate patients’ fatigue, so we decided to use the STAS-J.

Currently, however, no multidimensional scale allows both patients and health care providers to measure CRF. Therefore, we decided to use two scales to determine the degree of agreement between patients and health care providers in assessing severe malaise requiring care.

To evaluate the current CRF, the responses for each assessment tool were received simultaneously during the morning temperature check at the same time.

### Statistical analysis

All data were analyzed using easy-R version 1.61 statistical software. Statistical significance was set at *p* < 0.05. Descriptive statistics (frequency distribution, percentage, and means) were used to summarize the demographics. Spearman’s rank correlation coefficient was calculated to determine the association between CFS and STAS-J scores. The total CFS score was 19 or higher for the group with care needs, compared with the other observational group. Patients with a CFS score of 19 or more were underestimated if the nurse rated them as 1 or less on the STAS-J, overestimated if they had a CFS score of less than 19 and a STAS-J score of 2 or more, and the scores agreed if the above did not apply. Moreover, to clarify whether the duration of palliative care visits and the presence or absence of experience of receiving palliative care from nurses were related to the concordance of the ratings, we divided the duration of palliative care visits into two groups: less than 20 days and more than 20 days. Two groups of nurses met the paired patients for the first time and provided palliative care or did not provide it. The groups were then compared by performing Fisher’s exact test.

### Ethical issues

This study was approved by the ethics committee of Niigata University (Approval No. 2022-0090). Potential patient participants were identified by the treating palliative care physician and were provided with written information by a researcher explaining the study procedures. All participant patients and nurses were provided with information about written informed consent as well as about the option to withdraw from the study at any time. To obtain informed consent, the researcher approached each participant, and after explaining the purpose and procedures of the study, each participant signed a consent form and completed the questionnaire. Nurses who gave consent participated in a 30-minute orientation session on the use and precautions of the STAS-J and then completed the questionnaire.

## Results

A total of 32 individuals agreed to participate, of which 14 patients and 15 nurses completed the questionnaires as pairs, enabling inclusion in this study. A total of 28 participants, as 14 pairs, provided valid set responses, except for one nurse who could not be paired with a patient. Information on the total group of 28 patients and nurses is presented in Table 1.

**Table 1.** Characteristics of Participants

	Characteristics		*n*	%
Patients	Medical treatment type *n* = 14	Hospice	8	57.1
		Hospital	5	35.7
		Ambulatory patients	1	7.2
	Length admission in hospice (days) *n* = 8	<10	4	50.0
		11–20	1	12.5
		21–30	1	12.5
		31–40	1	12.5
		>41	1	12.5
	Length admission in hospital (days) *n* = 5	<10	1	20.0
		11–20	1	20.0
		21–30	2	40.0
		>31	1	20.0
	Length consultation in palliative care (days) *n* = 12	<10	3	25.1
		11–20	2	16.7
		21–30	1	8.3
		31–40	1	8.3
		41–50	1	8.3
		>51	4	33.3

		Mean ± SD	Minimum	Median	Maximum
	Length admission in hospice (days)	19.6 ± 16.7	5	13	50
	Length admission in hospital (days)	22.6 ± 11.9	4	24	35
	Length consultation in palliative care	58.9 ± 63.9	4	35	180
Nurse	Length of experience specialized palliative care (years) *n* = 14	<3	4	28.6	
		3–5	6	42.9	
		6–9	1	7.1	
		10–13	2	14.3	
		>14	1	7.1	
	Whether the paired patient has been giving care before *n* = 14	Yes	10	71.0	
		No	4	29.0	

The medical treatment types for the 14 patients enrolled in this study were hospice (57.1%), hospital (35.7%), and outpatient (7.2%). The lengths of consultation in palliative care were less than 20 days (41.8%) and 20 days or more (58.2%). The average time since consultation in palliative care was 58.9 (standard deviation [SD] = 63.9) days, with a range from 4 days to 180 days.

Among the 14 nurses, 4 (28.6%) had less than 3 years of experience in specialized palliative care, 7 (50.0%) had 3–9 years of experience, and 3 (21.4%) had 10 years or more of experience. Four (29.0%) nurses reported that they had never taken care of paired patients before.

### Patients’ self-assessment of fatigue

The mean for each CFS subscale data to assess the intensity of fatigue perceived by patients was Table 2. Total fatigue showed significant strong positive correlations with the physical fatigue (correlation coefficient r = 0.90, *p* < 0.001, 95% confidence interval [CI]: 0.71–0.91) and cognitive fatigue (r = 0.84, *p* < 0.001, 95% CI: 0.56–0.95) subscales, whereas it showed moderate positive correlation with affective fatigue (r = 0.57, *p* = 0.034, 95% CI: 0.05–0.84).

**Table 2.** Cancer Fatigue Scale Score in Patients (*n* = 14)

Factor	Score mean (SD)	Minimum	Median	Maximum
Total fatigue	24.4 (10.5)	9.0	26.5	39.0
Physical fatigue	11.7 (6.5)	1.0	11.0	21.0
Affective fatigue	7.6 (3.4)	4.0	7.0	15.0
Cognitive fatigue	5.1 (3.3)	0.0	6.0	11.0

The CFS total score showed that 8 (57.1%) of the 14 patients were classified into the care needs group and 6 (42.9%) into the observation group. The mean for each group was as follows: care needs group: total fatigue 32.5 (SD = 4.8), physical fatigue (subscale) 16.1 (SD = 4.3), affective fatigue 9.0 (SD = 3.4), and cognitive fatigue 7.4 (SD = 1.8); observation group: total fatigue 13.7 (SD = 3.7), physical fatigue 5.9 (SD = 3.2), affective fatigue 5.7 (SD = 2.4), and cognitive fatigue 2.2 (SD = 2.3).

### Nurses’ assessment of patient fatigue

The results of the nurses’ assessment of patient fatigue using the STAS-J showed that 8 nurses (57.1%) out of 14 were classified as nurses’ care needs, and 6 (42.9%) were classified as observation.

### Patients and nurses assessment agreement

**FIG. 1.**
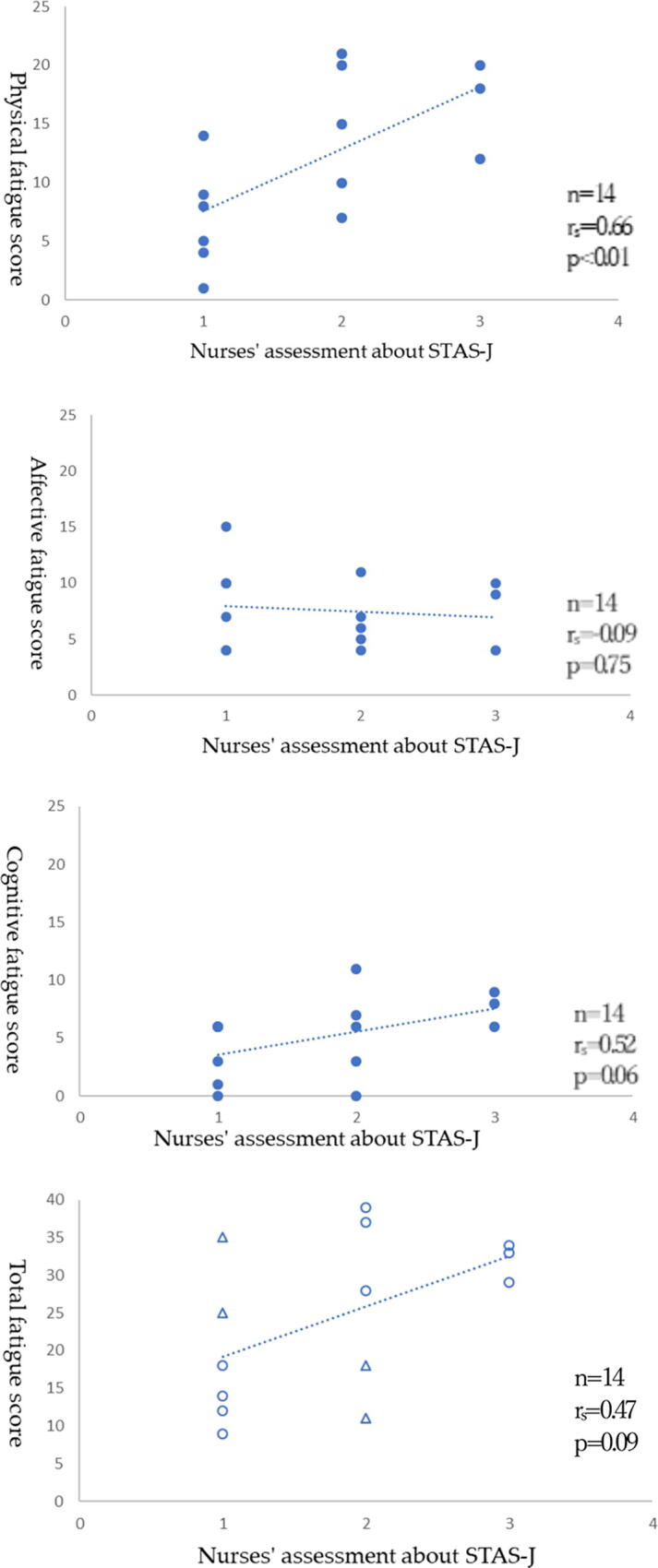
Distribution and association between CFS score and STAS-J score. Patients with a CFS score of 19 or more were underestimated if the nurse rated them as 1 or less on the STAS-J, overestimated if they had a CFS score of less than 19 and a STAS-J score of 2 or more, and agreed if the above did not apply. Total fatigue score and STAS-J score in the last figure, indicate agreement in assessments, and the dots indicate not agreements. CFS, the Cancer Fatigue Scale; STAS-J, the Support Team Assessment Schedule in Japan.

The fatigue assessment for care needs among the 14 pairs indicated agreement between patients and nurses in 10 pairs (71.4%), while 2 were underestimated and 2 (14.3%) were overestimated (Fig. 1 and Table 3).

**Table 3.** Patients and Nurses Assessment Agreement (14 pairs)

Patients’ and nurses’ assessment	Nurses’ assessment	Pairs	%
Agree		10	71.4
Not agree	Underestimated	2	14.3
	Overestimated	2	14.3

The correlation between each CFS subscale and the STAS-J was significant for total fatigue (rs = 0.47, *p* = 0.09), physical fatigue (rs = 0.66, *p* < 0.01), affective fatigue (rs =−0.09, *p* = 0.75), and cognitive fatigue (rs = 0.52, *p* = 0.06). Only physical fatigue showed a correlation between nurses’ and patients’ assessments.

Considering the difference in the agreement or incongruence in the ratings according to nurses’ experience of patients with care needs and the length of palliative care department visits, the results were as follows: nurses’ experience (*p* = 0.25, 95% CI: 0.00–3.81) and the length of palliative care department visits (*p* = 1.0, 95% CI: 0.01–17.23). There were no significant differences in agreement or incongruence ratings according to the presence or absence of experience or the length of palliative care department visits.

## Discussion

This study aimed to determine the congruence between patients’ and nurses’ assessment of patients’ fatigue at a clinically relevant end-of-life palliative care unit for terminally ill cancer patients. It also examined possible predictors of incongruence between nurses’ and patients’ ratings of patient fatigue. The results showed that more than half of the participant nurses agreed with their patients’ assessments, but even among those with experience with palliative care, more than one-fourth were incongruent. Among those who were incongruent, underestimation and overestimation of fatigue were equally distributed. In 2016, Loretta et al. reported that 14% of nurses underestimated the perception of fatigue evaluation in patients undergoing chemotherapy.^13^ Although the target patients were at different stages of illness, the underestimation rates were similar.

Underestimation has a more significant impact on patients than overestimation, as patients’ QoL is more likely to deteriorate without the necessary treatment and support. Since an underestimation may lead to a lack of support in their lives, increasing the consistency between patient and nurse assessments and basing assessments on patients’ subjective assessments and direct reports are important.^13,14^

One factor causing incongruence in fatigue evaluation may be the difference between the affective and cognitive subscales. The physical subscale can be evaluated from visual observations such as daily conversations and movements; however, affective and cognitive fatigue may be difficult for others to recognize and understand. This study revealed differences in the assessment of affective fatigue between patients and nurses. Patients with terminal cancer tend to experience spiritual pain, such as meaninglessness of life and values and emptiness.^10^ However, the *p* value between the cognitive subscale score and the STAS-J assessment was not significant, but the *p* value was 0.06, and the correlation coefficient was 0.52. Since the patients with cancer were at the very end of life, changes in their physical condition made it difficult for them to answer, even if they agreed to participate in the research. Therefore, the sample size assumed for this study could not be reached. Since the small sample size may not have resulted in significant differences, the sample size should be increased and the study should be conducted.

In addition, the patients and nurses may not often communicate about CRF. The duration of palliative care visits and the presence or absence of experience in receiving palliative care from nurses did not exhibit significant differences in the agreement or incongruence; it is possible that even though the experience of receiving palliative care was long, they did not have the opportunity to communicate about fatigue. Previous studies have found barriers to reporting fatigue among patients with cancer.^20–21^ This also requires nurses to reaffirm that it is difficult for patients to report fatigue to health care providers. Nurses need to build a trusting relationship with patients from the onset and try to create an environment in which patients can report various symptoms, including fatigue.

Gemma et al. reported that health care professionals lack confidence in assessing and completing an individualized management plan for patients with a life-limiting illness and distressing levels of fatigue.^22^ For health care providers to confidently assess fatigue and improve the accuracy of their assessments, educational programs on how to assess and manage fatigue should be implemented.

The study suggests that even nurses providing palliative care may not fully understand the symptoms of fatigue. It is highly important to make it a habit to ask the patients about their fatigue because fatigue is a subjective emotion and experience. Our study may lead to increased awareness of the need for the assessment of fatigue symptoms on an ongoing basis among patients with cancer in the clinic. Patient-reported outcomes through assessment may facilitate discussions between patients and the palliative care staff or team, which may lead to increased recognition of and targeted treatment for subjective symptoms such as fatigue. However, numerous terminally ill cancer patients are unable to self-report, thus there is a need to investigate the rating scales and factors to be used for subjects who are unable to self-evaluate.

In this study, it was difficult to know what the nurses were actually rating when they rated patients’ fatigue. Since the STAS-J was chosen based on the description attached to each step closest to the one used, the use of different scales did not allow us to determine which of the three subscales of fatigue ratings were in agreement or disagreement. A scale that can be used by both patients and health care providers must be developed in the future. Exploring whether they rely on daily movements to make judgments would also be interesting.

### Limitations

This study has several limitations that must be considered, including the difficulty of making patient–nurse pairs and answering the questions simultaneously. Since the study was conducted on patients at a single hospital in Japan, and its sample size was limited, making it difficult to generalize the results. The COVID-19 pandemic may also have contributed to the small sample size. However, despite this limitation, the agreement between the patients’ and nurses’ assessment of physical fatigue showed a significant difference, indicating that the nurses assessed accurately. Although the number of questions was limited to minimize the burden on patients, we believe that by clarifying the relationship between each symptom and each subscale, and in particular, the factors associated with affective fatigue, an assessment closer to the subjective evaluation of end-of-life patients with cancer can be made. In addition, we believe that examining the association with objective data such as body temperature and blood pressure, which are likely measured daily, may lead to an examination of methods for evaluating fatigue in patients who are unable to self-report. Future studies must not only include a larger sample size but also accumulate background factors of patients and nurses to clarify the factors affecting agreement or incongruence in the ratings. Another limitation of our study is that different scales were used to investigate the agreement between ratings for patients and nurses.

## Conclusions

Our study examined the congruence of fatigue evaluation between nurses and terminally ill cancer patients and found that more than half of the nurses were in congruence with the patients (10 cases out of 14). In the four cases of incongruence, two were underestimations and two were overestimations of fatigue. Differences in patients’ and nurses’ assessments were mainly regarding affective fatigue, which may have been under-assessed. As incongruent assessments may result in patients not receiving the support they need, health care providers must carefully consider affective fatigue.

## Abbreviations Used

CFS =: cancer fatigue scale
CRF =: cancer related fatigue
QOL =: quality of life
STAS-J =: Support Team Assessment Schedule in Japan
